# Intracellular signalling pathways and cytoskeletal functions converge on the psoriasis candidate gene CCHCR1 expressed at P-bodies and centrosomes

**DOI:** 10.1186/s12864-018-4810-y

**Published:** 2018-06-04

**Authors:** Mari H. Tervaniemi, Shintaro Katayama, Tiina Skoog, H. Annika Siitonen, Jyrki Vuola, Kristo Nuutila, Kristiina Tammimies, Sari Suomela, Esko Kankuri, Juha Kere, Outi Elomaa

**Affiliations:** 1 Folkhälsan Institute of Genetics, 00014 Helsinki, Finland; 2 Department of Medical and Clinical Genetics, Medicum and Research Programs Unit, Molecular Neurology, University of Helsinki, Helsinki, Finland; 3 Department of Biosciences and Nutrition, Karolinska Institutet, Huddinge, Sweden; 4 Helsinki Burn Center, Department of Plastic Surgery, University of Helsinki and Helsinki University Hospital, Helsinki, Finland; 5 Department of Pharmacology, Medicum, University of Helsinki, Helsinki, Finland; 6 Division of Neuropsychiatry, Department of Women’s and Children’s Health, Center of Neurodevelopmental Disorders, Karolinska Institutet, Stockholm, Sweden; 7 Department of Dermatology, University of Helsinki and Helsinki University Hospital, Helsinki, Finland; 8 School of Basic and Medical Biosciences, King’s College London, London, UK

**Keywords:** CCHCR1, PSORS1, Centrosome, Cytoskeleton, P-body, Cell adhesion, RNAseq, Expression profiling

## Abstract

**Background:**

*CCHCR1* (Coiled-Coil α-Helical Rod protein 1) is a putative psoriasis candidate gene with the risk alleles CCHCR1*WWCC and *Iso3, the latter inhibiting the translation of isoform 1. CCHCR1 was recently shown to be a centrosomal protein, as well as a component of cytoplasmic processing bodies (P-bodies) that regulate mRNA turnover. The function of *CCHCR1* has remained unsettled, partly because of the inconsistent findings; it has been shown to play a wide variety of roles in divergent processes, e.g., cell proliferation and steroidogenesis. Here we utilized RNA sequencing (RNAseq) using HEK293 cells overexpressing isoforms 1 or 3 (Iso1, Iso3 cells), in combination with the coding non-risk or risk (*WWCC) haplotype of *CCHCR1*. Our aim was to study the overall role of CCHCR1 and the effects of its variants.

**Results:**

The overexpression of CCHCR1 variants in HEK293 cells resulted in cell line-specific expression profiles though several similarities were observable. Overall the Iso1 and Iso3 cells showed a clear isoform-specific clustering as two separate groups, and the Non-risk and Risk cells often exhibited opposite effects. The RNAseq supported a role for CCHCR1 in the centrosomes and P-bodies; the most highlighted pathways included *regulation of cytoskeleton*, *adherens* and *tight junctions*, *mRNA surveillance* and *RNA transport*. Interestingly, both the RNAseq and immunofluorescent localization revealed variant-specific differences for CCHCR1 within the P-bodies.

**Conclusions:**

CCHCR1 influenced a wide variety of signaling pathways, which could reflect its active role in the P-bodies and centrosomes that both are linked to the cytoskeleton; as a centrosomal P-body protein CCHCR1 may regulate diverse cytoskeleton-mediated functions, such as cell adhesion and -division. The present findings may explain the previous inconsistent observations about the functions of CCHCR1.

**Electronic supplementary material:**

The online version of this article (10.1186/s12864-018-4810-y) contains supplementary material, which is available to authorized users.

## Background

Psoriasis is a chronic multifactorial dermatological disorder, characterized by abnormal proliferation and differentiation of keratinocytes and infiltration of inflammatory cells. There are several susceptibility gene loci for psoriasis (*PSORS1*–*15*) (based on the Online Mendelian Inheritance in Man, OMIM); of which *PSORS1* (6p21.3) has the strongest risk effect [1]. Diverse psoriasis-associated alleles have been identified within the region. However, a strong linkage disequilibrium has made it difficult to distinguish their individual effects. Hence, the effector genes in psoriasis within the 6p21.3 region are currently not fully understood. *CCHCR1* (Coiled-Coil α-Helical Rod protein 1) is a putative *PSORS1* candidate gene among others [2–4], and its CCHCR1*WWCC allele is associated with psoriasis in several populations [2, 3, 5]. WWCC stands for the amino acids in the psoriasis risk haplotype, whereas in the non-risk haplotype the corresponding amino acids are RRGS. We have previously described a novel form of CCHCR1, isoform 1, where the N-terminal domain is longer than in isoform 3 [6]. The formation of isoform 1 is dependent on a SNP (rs3130453) that results in either a longer open reading frame (allele *Iso1) or a stop codon (allele *Iso3), enabling only the translation of isoform 3. In addition, there are two transcription start sites, only one of them enabling the production of long isoform 1. Notably, we demonstrated that *Iso3 shows association with psoriasis (*P* < 10^− 7^) [6].

The CCHCR1 protein does not belong to any known protein family but is predicted to be a rod-like protein, with an alpha-helical coiled coil structure. Although its expression pattern in psoriatic skin differs from healthy skin [7, 8] and its overexpression influences cell proliferation [6, 9, 10], its role as a putative psoriasis effector gene has remained unsettled, partially because it has been suggested to function in several divergent biological processes such as the regulation of steroidogenesis via mitochondrial steroidogenic activator protein (STAR) [8, 11] or muscle differentiation via RNA polymerase II subunit 3 (RPB3) [12]. We have also demonstrated that in addition to its cytoplasmic location, both CCHCR1 isoforms localize at the centrosome [6], a cell organelle playing a crucial role in cell division. The centrosomal localization of CCHCR1 has been verified in other large-scale proteomics studies [13, 14]. Furthermore, CCHCR1 has been shown to interact with mitotic spindle proteins [15], providing evidence for its possible involvement in cell division. A recent study demonstrated that, in addition to its location at the centrosome, CCHCR1 is a component of processing bodies (P-bodies) [15]. P-bodies are cytoplasmic ribonucleoprotein granules that regulate mRNA turnover in a post-transcriptional manner and are involved in mRNA degradation, surveillance, and transport, translational repression, and RNA-mediated gene silencing [16, 17]. There is a link between the P-bodies and centrosomes; P-bodies transit along microtubule tracks to and from the centrosome and furthermore, a pair of stationary P-bodies reside at the centrosome during the interphase [18, 19].

Using stable HEK293 cells overexpressing isoforms 1 or 3 with the non-risk (*RRGS) or the risk haplotype (*WWCC) we have shown that isoform 3 accelerates cell proliferation and together with the *WWCC allele apoptosis as well. Whereas isoform 1 lacks significant effects on cell proliferation or cell cycle progression. Furthermore, the CCHCR1-HEK293 cell lines show isoform- and haplotype-specific changes in cell size and shape and have alterations in the organization and expression of the cytoskeletal proteins actin, vimentin, and cytokeratins. We also demonstrated that CCHCR1 may regulate EGF-induced STAT3 activation in an isoform-specific manner [6].

Here we applied 5’end-targeted RNA sequencing (RNAseq) on the previously established and characterized CCHCR1-HEK293 cell lines (Iso1Risk, Iso1Non-risk, Iso3Risk, and Iso3Non-risk) [6, 20] to examine isoform- and haplotype-specific effects on global gene expression profiles, and to identify the underlying mechanisms leading to the previously observed differences between the CCHCR1 cell lines. The sensitivity of RNAseq allowed an in-depth assessment of altered gene expression in the cell lines, making it possible to get a better understanding of the effects of CCHCR1 isoforms on the cellular pathways.

## Results

### CCHCR1-HEK293 cell lines exhibited isoform- and haplotype-specific gene expression profiles

In the present study we used stably transfected CCHCR1 cell lines that were previously generated into human embryonic kidney (HEK) 293 cells. HEK293 is an adenovirus-transformed cell line that is considered tumorigenic. The establishment and characterization of the CCHCR1 overexpressing HEK293 clones have been described previously [6]. Briefly, the CCHCR1 cell lines (Iso1Risk, Iso1Non-risk, Iso3Risk, and Iso3Non-risk) were screened for the CCHCR1 expression by qPCR, fluorescence microscopy, and western blotting. The clones used in the present work were chosen based on the previous studies; we selected clones with similar CCHCR1 expression levels and cell morphology. As most of the previous CCHCR1 studies have focused on isoform 3, we first performed gene expression profiling using Affymetrix Human Gene ST 1.0 microarrays for the Iso1Non-risk and Iso1Risk CCHCR1 cell lines to increase knowledge of isoform 1. Wild type HEK293 and vector-transfected cells were used as controls in the comparative expression analysis. The comparison of Iso1Non-risk and Iso1Risk with the vector control revealed 296 and 206 differentially expressed genes (DEGs) (Cut-off fold change1.3-fold, *p* < 0.002), respectively (Additional file 1: Table S1). Gene enrichment analyses (the Database for Annotation, Visualization and Integrated Discovery (DAVID) [21, 22], WebGestalt 2013, and WebGestaltR 2017 [23]) of the DEGs (> 1.5-fold) from the Iso1Non-risk cells revealed such pathways and functions (*p* < 1 × 10^− 4^) as *focal adhesion*, *ECM-receptor interaction*, and *regulation of actin cytoskeleton*. The results of the gene enrichment analysis are shown in detail in Additional file 1: Table S1. Though the microarray study suggested differences in the gene expression between Iso1Non-risk and Iso1Risk, the cell lines sharing only 50 genes, the sensitivity of the expression profiling remained rather modest. Particularly in the Iso1Risk cell line only a few pathways, such as *cell cycle*, *focal adhesion*, and *leukocyte migration* showed enrichment of more than four genes when WebGestalt was used. To get a higher sensitivity assessment of altered gene expression in all four CCHCR1-HEK293 cell lines we performed RNAseq.

**Fig. 1:**
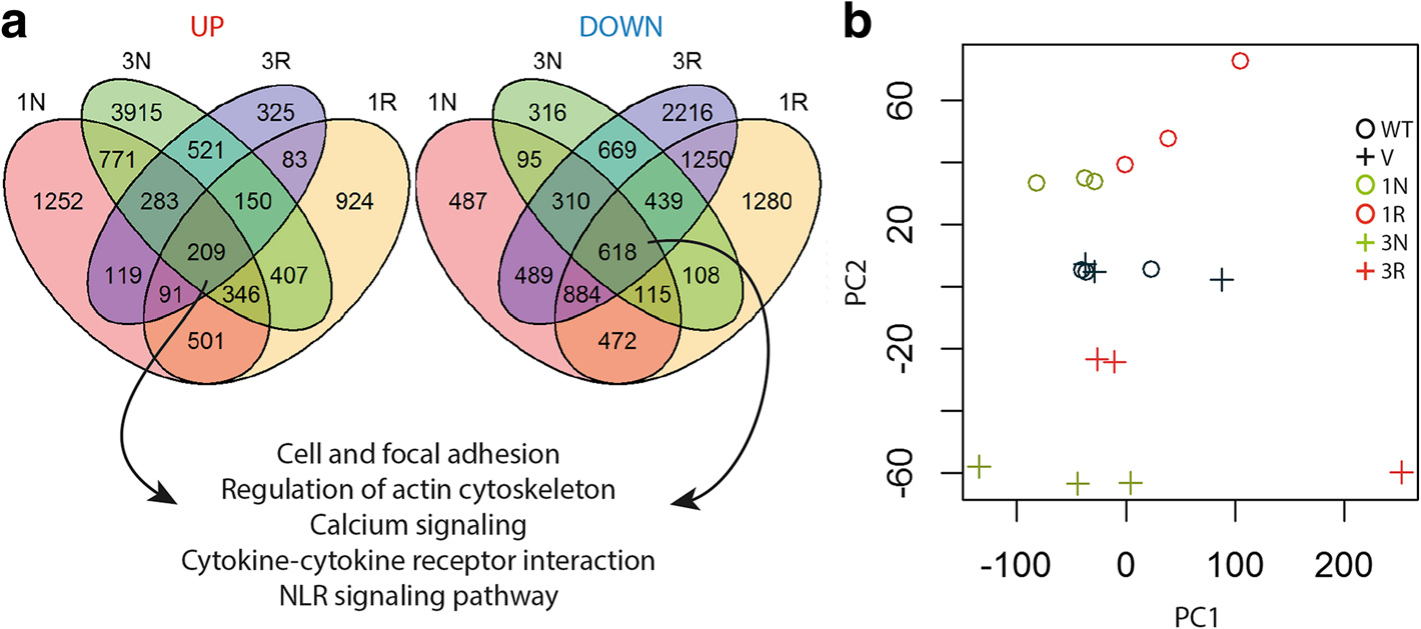
Expression profiles of the CCHCR1-HEK293 cell lines. **a** Venn diagrams of differentially expressed genes (DEGs) of the CCHCR1-HEK293 cell lines; Iso1Non-risk (1 N), Iso1Risk (1R), Iso3Non-risk (3 N), and Iso3Risk (3R), when compared with vector and wild type controls (FC > 1.5 or FC < 0.75, FDR < 0.25). The most highlighted functions and pathways of the gene enrichment analysis of DEGs that were shared by the all four cell lines are shown. Up- and downregulated genes were analyzed separately (see in detail Additional file 3: Table S3). **b** Principal component analysis (PCA) demonstrates differences between CCHCR1-HEK293 cell lines. The two PCs depict the variation between samples. The PC2 illustrates the isoform-dependent difference, clustering isoforms 1 and 3 as separate groups. The wild type (wt) and vector (v) control samples cluster as an overlapping group between the isoform 1 and isoform 3 samples

RNA samples extracted from the wild type HEK293, the vector control, and the CCHCR1 cell lines (Iso1Non-risk, Iso1Risk, Iso3Non-risk, and Iso3Risk), were subjected to RNAseq. The comparisons of transcripts from each CCHCR1-HEK293 cell line with the controls (wild type and vector) revealed a large number of differentially expressed genes (DEGs) that are listed in Additional file 2: Table S2. The number of upregulated genes was highest in Iso3Non-risk (6602) and lowest in Iso3Risk (1781), while the number of downregulated genes was highest in Iso3Risk (6968) and lowest in Iso3Non-risk (2806) (up: FC > 1.5, down: FC < 0.75, FDR 0.25). All the CCHCR1 cell lines shared 209 upregulated and 618 downregulated genes (Venn diagram of DEGs shown in Fig. 1a).

There were notable differences in the expression profiles between the CCHCR1-HEK293 cell lines. We estimated the dissimilarity between the cell lines by principal component analysis (PCA) (Fig. 1b) that enabled multiclass comparisons between samples [24]. The second PC (PC2) depicts the isoform-dependent difference, clustering isoform 1 (Iso1) and 3 (Iso3) as separate groups. The wild type and vector control samples clustered as an overlapping group between the Iso1 and Iso3 samples. The PCA demonstrated that the replicates differed to some extent, especially the Iso3Risk samples (the outlier specimen 3Rc, Fig. 1b). The clustering of Risk and Non-risk samples as separate groups was less clear than observed with Iso1 and Iso3.

### Expression profiling of CCHCR1-HEK293 cell lines highlighted cell adhesion and regulation of cytoskeleton

Using the WebGestalt tool (WebGestalt 2013 and WebGestaltR) for gene enrichment analysis, we analyzed the DEGs of four different comparisons (DEGs shown in Additional file 2: Table S2: Iso1Non-risk, Iso1Risk, Iso3Non-risk, and Iso3Risk cells compared with wild type and vector controls). The KEGG pathway analysis of up- and downregulated genes revealed several similarities between the CCHCR1-HEK293 cell lines (Additional file 3: Table S3 showing the results of gene enrichment analysis). Adhesion-related pathways, such as *focal adhesion*, *gap junction*, *tight junction*, *adherens junction*, and *regulation of actin cytoskeleton*, as well as *Wnt signaling* and *calcium signaling*, were highlighted in all the cell lines. In addition, *MAPK*, *ErbB*, and *TGF-beta* were among the top pathways. Their expression profiles, however, showed variation between the control samples. The most highly up- and downregulated genes (1 × 10^8^ < FC < 1 × 10^− 7^) were enriched especially in functions and pathways that are involved in *neuroactive ligand–receptor interaction*, *cell adhesion*, *calcium signaling*, and *cytokine-cytokine receptor interaction* (Additional file 3: Table S3).

**Fig. 2:**
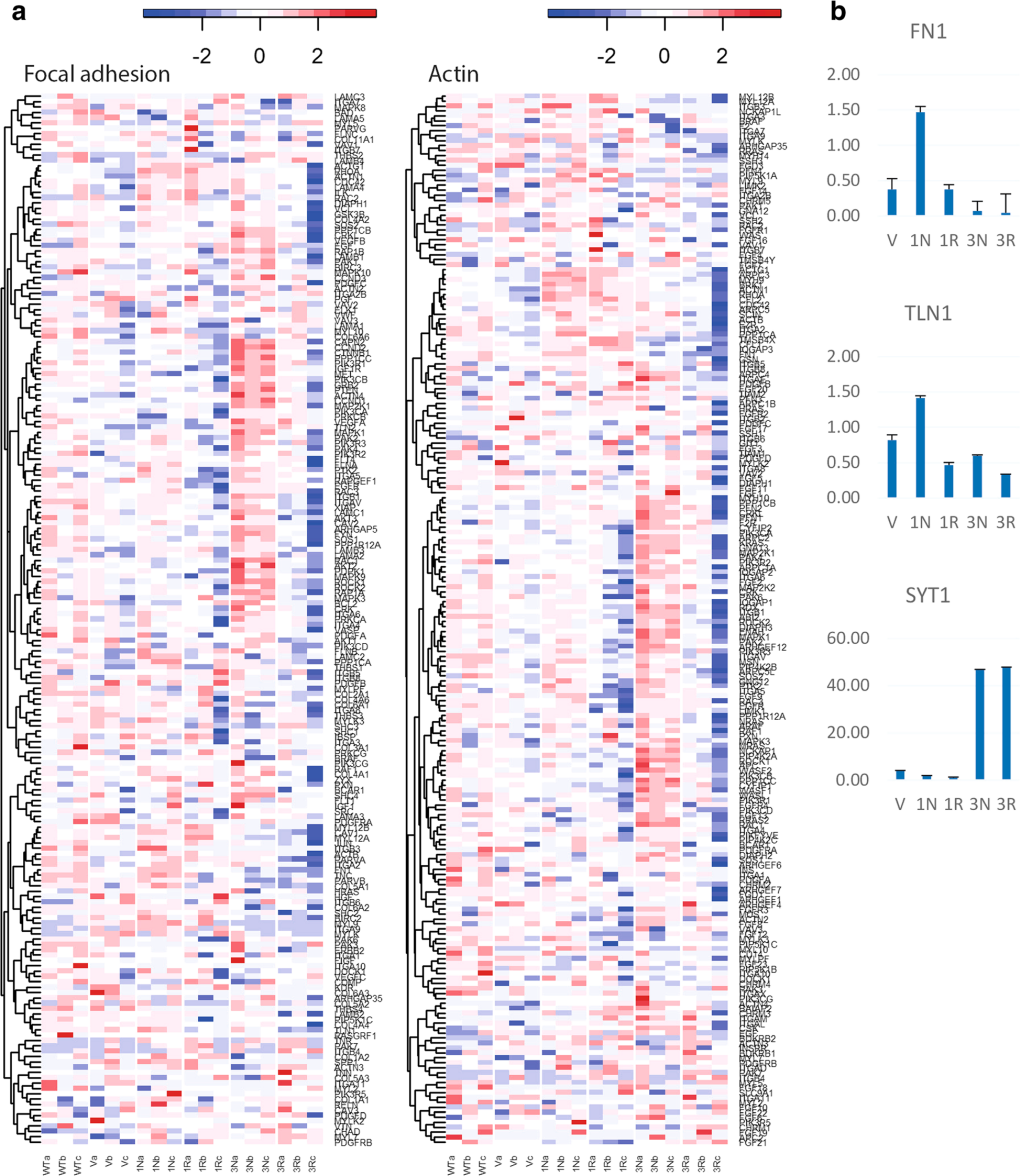
Expression profiling of the CCHCR1-HEK293 cell lines highlighted cell adhesion and actin cytoskeleton. **a** Heatmaps showing gene expression of *focal adhesion* and *regulation of actin cytoskeleton* in the CCHCR1 Iso1Non-risk (1 N), Iso1Risk (1R), Iso3Non-risk (3 N), and Iso3Risk (3R) cell lines, and in the vector transfected cells (V), and wild type HEK293 (WT). Color key: red represents upregulated and blue downregulated expression (row Z-score). **b** The expression of *FN1*, *TLN1*, and *SYT1* in the CCHCR1-HEK293 cell lines was validated with qPCR, and compared with the gene expression in the wild type cells. Error bars: standard deviation. Here shown normalization with *HPRT1*

Although the same pathways were showing enrichment in all the CCHCR1-HEK293 cell lines, their gene expression profiles differed; different genes were highlighted, and the gene expression varied from down- to upregulation between the cell lines. For example, *focal adhesion* and *regulation of actin cytoskeleton* were the top pathways in all the lines (Additional file 3: Table S3). However, the corresponding heatmaps showed differences between the cell lines; the Non-risk and Risk cells often exhibited opposite effects (Fig. 2a showing heatmaps of *focal adhesion* and *regulation of actin cytoskeleton*). Several relevant genes of *focal adhesion* and *actin cytoskeleton* were downregulated particularly in the Iso3Risk cells. To verify this, we quantified the expression of *TLN1* (talin 1) and *FN1* (fibronectin 1) by qPCR (shown in Fig. 2b). They were highly downregulated in both isoform 3 expressing cell lines (Iso3), especially in Iso3Risk, whereas their expression was increased in the Iso1Non-risk cells similarly as was observed in RNAseq. We also examined the expression of *SYT1* (synaptotagmin 1) by qPCR (Fig. 2b); one of the most highly upregulated genes in the Iso3 cells. The qPCR verified that its expression was increased only in the Iso3 cells and was unaltered in both isoform 1 expressing cells (Iso1). Overall, the qPCR verification results agreed with the RNAseq data. We have previously shown that our RNAseq protocol enables the accurate quantitation of gene expression [24].

To study the overall biological role of CCHCR1, we analyzed the DEGs that were shared by the four CCHCR1-HEK293 cell lines (827 genes, 0.75 > FC > 1.5, FDR < 0.25). The analyses of shared DEGs (Additional file 3: Table S3: Intersection up and down) identified such pathways and genes as: *calcium signaling* (*PLCD1*, *PLCB2*), *NOD-like receptor signaling* (*NLRP3*, *CARD9*, and *CXCL1*), *cytokine-cytokine receptor interaction* (*IL12A*, *IL22RA1*, and *VEGFC*), *cell adhesion* (*ITGAL*, *CNTN1*, and *CLDN7*), and *regulation of actin cytoskeleton* (*PAK3*, *RRAS*, and *ITGAX*), however, the gene counts of the detected pathways were modest.

### Isoform- and haplotype-specific effects of CCHCR1 on cell signaling

**Fig. 3:**
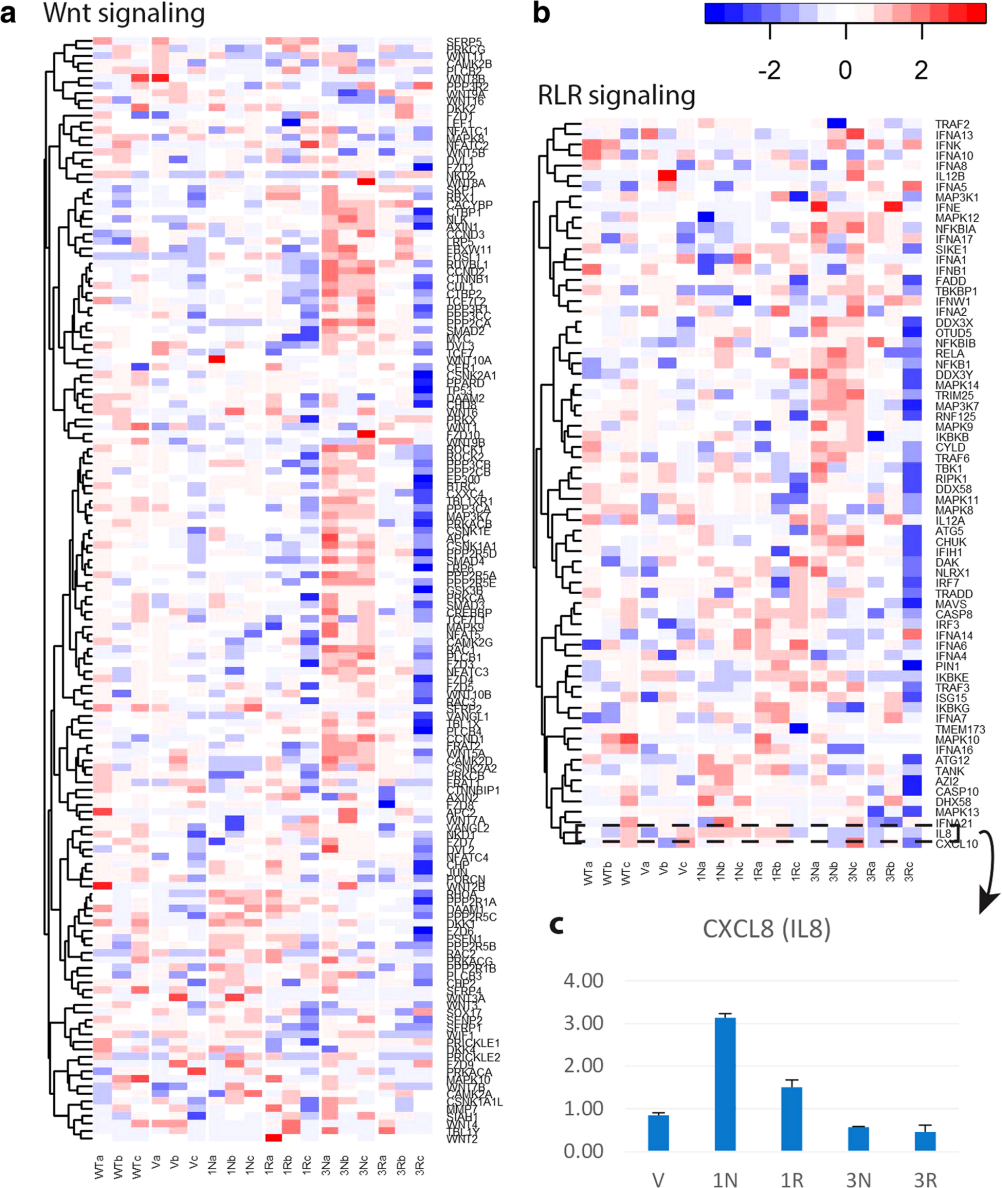
Gene expression profiles of Wnt and RIG-I-like signaling in the CCHCR1-HEK293 cell lines. Both pathways were highlighted in the CCHCR1 cell lines. Heatmaps of Wnt (**a**) and RIG-I-like (RLR) (**b**) signaling in the Iso1Non-risk (IN), Iso1Risk (1R), Iso3Non-risk (3 N), and Iso3Risk (3R) CCHCR1 cell lines, vector transfected cells (v), and wild type HEK293 (wt) demonstrated that there were differences between cell lines. Wnt signaling is mainly upregulated only in the Iso3Non-risk cells while RLR signaling shows more downregulation in Iso3Risk than in other cell lines. Color key: red represents upregulated and blue downregulated expression (row Z-score). **c** The expression of *CXCL8* in the CCHCR1-HEK293 cell lines was validated with qPCR, and compared with the gene expression in the wild type cells. Here shown normalization with *HPRT1*. Error bars: standard deviation

Despite similarities between the CCHCR1-HEK293 cell lines, some isoform- and haplotype-specific effects on the gene expression were apparent, which is observable from the Venn diagram (Fig. 1a) and the heatmaps. For instance *Wnt signaling* (heatmap in Fig. 3a) was mainly upregulated only in the Iso3Non-risk cells when compared with controls (Additional file 3: Table S3). While several pathways were mainly downregulated in the Iso3Risk cells, these including *regulation of actin cytoskeleton* (heatmap Fig. 2a) and *RIG-I-like receptor signaling* (heatmap in Fig. 3b; Additional file 3: Table S3). As *CXCL8* (alias *IL8*) is involved in several signaling pathways that were highlighted in our expression data, as well as related to psoriasis, we validated its expression in the CCHCR1-HEK293 cell lines by qPCR; *IL8* showed reduced expression in the Iso3 cell lines and upregulation in the Iso1cells, especially in Iso1Non-risk (Fig. 3c), as observed in RNAseq.

**Fig. 4:**
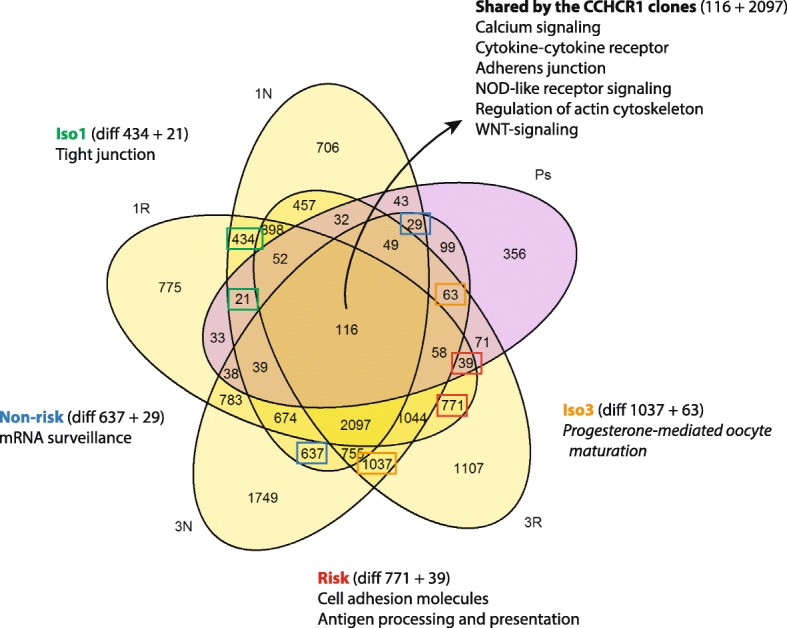
Isoform- and haplotype-specific differences between the CCHCR1-HEK293 cell lines. The Venn diagram of the DEGs (FC > 1.5 or < 0.75, FDR < 0.25) from the CCHCR1-HEK293 cell lines Iso1Non-risk (1 N), Iso1Risk (1R), Iso3Non-risk (3 N), and Iso3Risk (3R) when compared with the control cells, and the DEGs from the psoriatic skin samples (Ps) [20]. The most highlighted gene enrichment pathways of DEGs (both the up- and down-regulated) that were shared by the Non-risk, Risk, Iso1, Iso3, or all the CCHCR1 cell lines are shown. The DEGs used for the gene enrichment analysis are shown in parentheses and indicated by color coded rectangles in the diagram. The DEGs (diff) and the results are shown in detail in the Additional file 4: Supplement Table S4

To study the isoform and haplotype-specific effects of CCHCR1 more specifically, and to get more statistical power for the study, we analyzed the DEGs in four different groups; DEGs shared by the Iso1, Iso3, Non-risk, or Risk cell lines (Fig. 4: the Venn diagram of comparisons). The DAVID and WebGestalt (versions 2013 and 2017) analyses revealed differences between the groups (Additional file 4: Table S4 showing gene enrich analysis in detail), though the number of gene counts in highlighted pathways remained modest. *Tight junction* was highlighted in the shared DEGs of the Iso1 cells (FDR = 0.06), *progesterone mediated oocyte maturation* in the Iso3 cells (FDR < 0.05), *mRNA surveillance pathway* in the Non-risk cells (FDR = 0.02), and *antigen processing and presentation* in the Risk cell lines (FDR < 0.05). The gene enrichment analysis of DEGs including both the up-and downregulated genes that were shared by all the CCHCR1-HEK293 cell lines (Fig. 4 Venn diagram) highlighted only a few pathways with FDR < 0.1 when analysed by WebGestaltR, these including *calcium-* and *estrogen signaling* (FDR = 0.05). WebGestalt highlighted *cytokine-cytokine receptor interaction*, *NOD-like receptor signaling*, and *adherens junction* as well.

As the Iso1 and Iso3 cell lines showed a clear isoform-specific clustering in the PCA (Fig. 1b), we pooled the Iso1Non-risk and Risk samples and compared with the pooled vector and wildtype data (comb_Iso1), and similarly we pooled and compared Iso3Non-risk and Risk vs vector and wildtype (comb_Iso3) to increase the number of replicates and to get more statistical power for the study. The DEGs were re-extracted by a two class unpaired comparison using a higher permutation (*n* = 1000). (Additional file 5: Table S5 showing the DEGs and the results of gene enrichment analysis for comb_Iso1 and comb_iso3). We also pooled the data from Iso1Non-risk and Iso3Non-risk (comb_Non-risk), and from Iso1Risk and Iso3Risk (comb_Risk) (Additional file 6: Table S6 showing the DEGs and the results of gene enrichment analysis for comb_Non-risk and comb_Risk), although the haplotypes showed less clustering than the isoforms. To control the specificity of the results we also generated mock gene lists by permuting the sample groupings in the comparison (Additional file 6: Table S6 showing the summary of gene enrichment analyses of the mock gene lists). Several pathways were highlighted among the most mock gene lists. There were, however, pathways that were highlighted specifically among the CCHCR1-HEK293 clones; *adherens junction* and *tight junction* in comb_Iso1, and *RNA transport* and *mRNA surveillance* in comb_Iso3 (FDR < 0.002) and comb_Non-Risk (FDR = 0.01) (Additional file 5: Table S5; Additional file 6: Table S6), which agreed the results obtained with the cell line specific DEGs (Additional file 3: Table S3). *RIG-I-like receptor signaling*, which was highlighted in the pathway analyses of individual CCHCR1 clones (Additional file 3: Table S3), rose up in comb_Iso1 (FDR = 0.06) as well. *Progesterone mediated oocyte maturation* was highlighted in comb_Iso3 (FDR = 0.01) as was also detected in the analysis of the shared Iso3 DEGs (Additional file 4: Table S4; Additional file 5: Table S5). *Mitochondria* that play a role in the sythesisof steroids was highlighted among the comb_Iso3 DEGs as well. Several *HLA* genes were overrepresented especially in comb_Iso3, highlighting pathways such as *systemic lupus erythematosus* (FDR = 0.0002) and *antigen processing and presentation* (FDR = 0.02)*.* The latter and *HLA*-genes were highlighted also in the pathways analysis of comb_Risk which otherwise showed much less overrepresented pathways than the other groups.

### Gene expression in the CCHCR1-HEK293 cell lines and psoriatic skin

The comparison of transcriptomes from two completely different biological systems, such as the CCHCR1-HEK293 cell lines and psoriatic skin, is challenging and the direct comparison of gene expression changes is unfeasible. Some similarities were observable when the current expression results of CCHCR1 cells were compared to our previous RNAseq data on psoriatic skin obtained from the comparison between psoriatic lesional and non-sessional skin, and healthy control skin [20]. The comparison revealed some similarities in the gene expression changes, such as upregulation of *S100* and *KRT* (*KRT6*, *KRT16*, and *KRT17*) genes, and enrichment of the steroid-related pathways. Some genes of *RIG-I-like receptor signaling* (*DDX58*, *CXCL8,* and *CASP8*) showed altered expression in the CCHCR1-HEK293 cells and psoriatic skin, as well. The results, however, should not be over interpreted, as psoriasis is a complex disease and CCHCR1 is a susceptibility gene among several others, and as mentioned the direct comparison of CCHCR1 cells and skin samples is not warranted.

We also examined the CCHCR1 genotype (*Iso1 or *Iso3, *Non-risk or *Risk allele) and the most well-known psoriasis haplotype HLA-Cw*06:02 in our psoriatic and healthy skin samples. The genotyping results are shown in detail in Additional file 7: Table S7. We genotyped the SNPs for rs3130453 (G/A) resulting *Iso1 or *Iso3 allele and rs130076 (C/T) corresponding to the second R/W amino acid change in CCHCR1*WWCC risk allele. Five out of seven psoriatic samples were homozygous for the *Iso3 allele, whereas only two out of eight were homozygous in the controls. None of the psoriatic samples had the homozygous *Iso1 genotype. Only two of the psoriatic patients were heterozygous and therefore able to translate the longer isoform 1 as well. Five out of seven psoriatic samples were heterozygous for the Risk (*WWCC) haplotype whereas five out of eight controls had the homozygous Non-risk haplotype. Most of the control samples were negative for the psoriasis haplotype HLA-Cw*06:02 (six out of eight) but most of the psoriasis samples were heterozygous positive (five out of seven). The genotyping of psoriatic samples agrees the previous results [20], but the number of samples didn’t allow studying the correlation of CCHCR1 genotype and gene expression profiles. The systematic identification of all potentially causal variants in psoriasis in order to study the effects of individual variants or their combinations would be an extremely laborious task to implement in practice. The CCHCR1 and HLA-Cw6 genotypes were illustrated in a PCA plot that clusters the sample types (Additional file 7: Figure S2).

### CCHCR1 affects P-body-related functions and shows isoform-specific localization with P-bodies

**Fig. 5:**
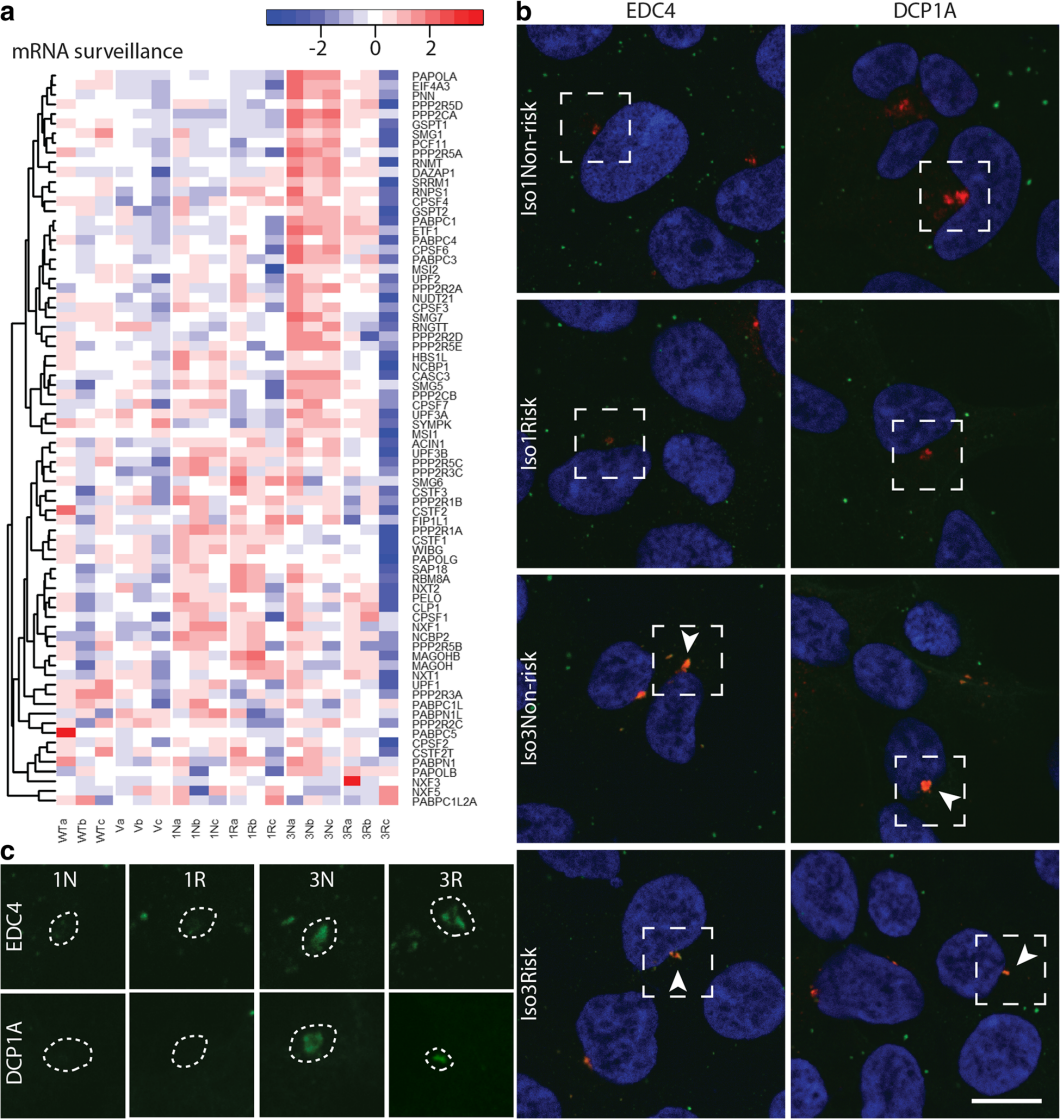
CCHCR1 shows isoform-specific localization with centrosomal P-bodies and affects mRNA surveillance pathway. **a** Heatmap showing gene expression profiles of mRNA surveillance pathway in Iso1Non-risk (1 N), Iso1Risk (1R), Iso3Non-risk (3 N), and Iso3Risk (3R) CCHCR1-HEK293 cell lines, and in control cells; vector (V) and wildtype (WT). Color key: red represents upregulated and blue downregulated expression (row Z-score). **b** Immunofluorescent staining of the CCHCR1 (red) cell lines: Iso1Non-risk, Iso1Risk, Iso3Non-risk, and Iso3Risk, with the P-body markers (green) EDC4 and DCP1A. Panel **b** shows the overlap of red and green, and panel **c** shows the green channel images of the corresponding sites. The colocalization (orange) of CCHCR1 and P-body markers is absent in the Iso1Non-risk and Iso1Risk cells: only the CCHCR1 expression (red) is observable at the centrosomes and cytoplasm (**b**), whereas the antibodies against EDC4 and DCP1A fail to recognize these structures (**c**). In Iso3Non-risk and Iso3Risk, the colocalization (orange, marked with arrow heads in panel **b**) is observable with antibodies against the EDC4 and DCP1A (green in panel **c**). Nuclei are stained with DAPI (blue). Scale bar: 10 μm

The recent study [15] that localized CCHCR1 at P-bodies was carried out with the Iso3Non-risk form of CCHCR1. Interestingly in our RNAseq data, the P-body-related pathways *RNA transport*, *RNA degradation*, and *mRNA surveillance* were highlighted in the Iso3 cells (WebGestalt *p* < 1 × 10^− 15^) and especially in the Non-risk cells (FDR < 0.1) (Additional file 5: Table S5; Additional file 6: Table S6). Also the comparison Iso3Non-risk cells versus controls revealed the enrichment of mRNA surveillance (FDR = 1.7 × 10^− 9^) (Additional file 3: Table S3: the gene enrichment results of 3 N); several *SMG* genes (e.g. *SMG1*, *SMG5*, and *SMG7*) that code nonsense mediated mRNA decay factors of surveillance complex [25], and *CPSF* genes encoding cleavage and polyadenylation specific factors were upregulated (1.5 < FC < 2.5). Heatmap of *mRNA surveillance* gene expression in the CCHCR1-HEK293 cell lines is shown in Fig. 5a. Although the gene expression changes were moderate, more than half of the genes acting in the mRNA surveillance pathway showed altered expression in Iso3Non-risk (the KEGG pathway of *mRNA surveillance* is shown in detail in Additional file 3: Table S3: mRNA surveillance).

Here, we studied by immunofluorescence staining with the P-body markers EDC4 and DCP1A (Fig. 5b, c) [15] and the centrosomal marker γ-tubulin (Additional file 8: Supplementary information Figure S2) whether the different CCHCR1 isoforms localize with the P-bodies in the same manner. We observed differences in the staining pattern between the isoforms; in the Iso1 cells, especially in Iso1Non-risk, the P-body marker colocalized rarely with CCHCR1, whereas in the Iso3 cells the P-body staining colocalized with the CCHCR1 granules (*P* < 4 × 10^− 19^) (Additional file 8: Supplementary information describes the counting of colocalization of CCHCR1 and P-body markers). In addition, we demonstrated by γ-tubulin staining that in the Iso1 cells P-body markers showed less accumulation at the centrosomes (Additional file 8: Figure S2).

## Discussion

**Fig. 6:**
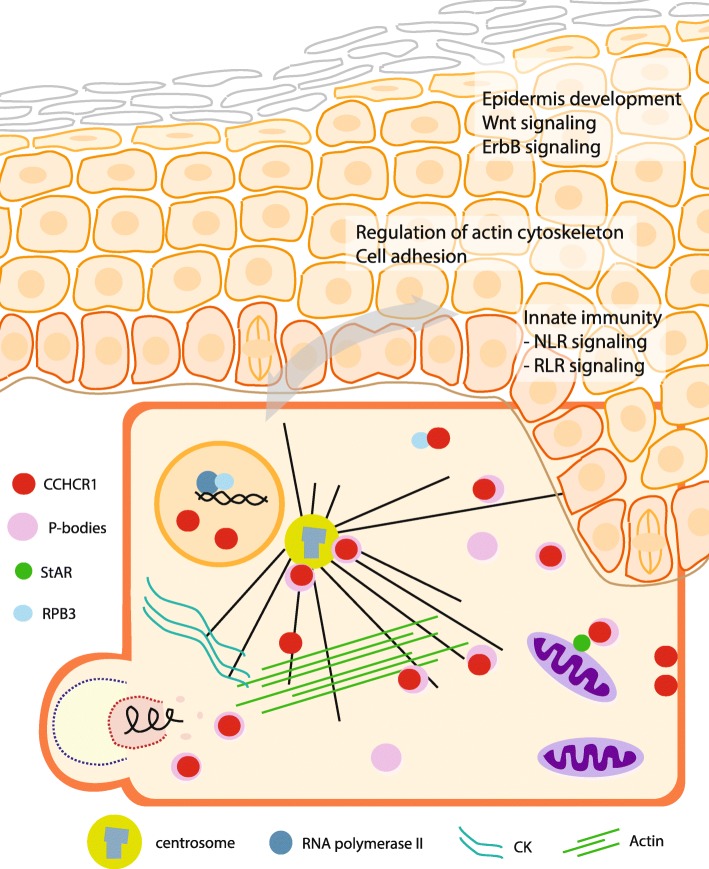
Model for CCHCR1 action in a cell and skin. CCHCR1 is expressed in a basal layer of keratinocytes in the epidermis of the healthy skin. In the thickened psoriatic epidermis, it is expressed in supra-basal layers as well. It is a dynamic protein that might play a role both in the cytoplasm at the centrosomes and P-bodies that occasionally colocalize, and possibly in the nucleus as well. Both centrosomes and P-bodies are physically linked to the cytoskeleton (cytokeratin (CK), actin, and microtubules (black lines)) that functions as a scaffold for the regulation of e.g. cell division and adhesion, mRNA transport and turnover, and transport of organelles like mitochondria. The previous results and the present RNAseq suggested that CCHCR1 regulates expression of cytoskeletal components and functions related to cytoskeleton, such as cell adhesion [6]. Furthermore, CCHCR1 may control the mRNA surveillance via the P-bodies, which may affect a wide variety of biological processes. Previous studies have identified several interaction partners for CCHCR1, these including the mitochondrial StAR protein that regulates steroidogenesis [8, 11], the RNA polymerase II subunit 3 (RPB3) that regulates transcription [12], some mitotic spindle proteins [15], and several viral proteins [30]. These interactions may result from the P-body localization of CCHCR1, or from the dynamic nature of CCHCR1 and its ability to regulate various processes via cytoskeleton. RNAseq identified several pathways for CCHCR1 which are relevant for the pathogenesis of psoriatic epidermis, epidermis development, and innate immunity. Interestingly, also the innate immunity including the RIG-like receptor (RLR) signaling is affected by the P-bodies

Our results show the power of RNAseq providing a large amount of detailed expression data on the HEK293 cells expressing the CCHCR1 variants. The principal component and gene enrichment analyses demonstrated that the cell lines expressing different isoforms and haplotypes of CCHCR1 have their distinct, specific expression profiles. The approach also strengthened the previous findings of the putative roles of CCHCR1 in the regulation of cytoskeleton (Fig. 6) and steroid biosynthesis [6, 8]. Additionally, the RNAseq revealed that CCHCR1 affected the cell signaling pathways mediated via the P-bodies, which provided evidence of its possible active role at the P-bodies. Furthermore, we observed isoform- and haplotype-specific differences in its effects on the P-body-related functions, as well as on the localization with the P-bodies. The P-body localization may also explain some of the putative interactions of CCHCR1 that have been previously identified with such proteins as StAR, RPB3, and viral proteins (Fig. 6) [11, 12, 26]. We also compared the gene expression results of the CCHCR1-HEK293 cell lines and psoriatic skin, though the direct comparison of transcriptomes of two completely different biological sample types is unfeasible. The approach was unable to give any final direct answers to the question what is the putative role of different CCHCR1 variants. As an adenovirus-transformed cell line HEK293 cells have gone through structural alterations of genome. Therefore it is possible that we have missed some gene expression data of the CCHCR1 cell lines. Furthermore the tumorigenic properties of HEK293 may have affected the gene regulation and -expression of the cell lines.

### CCHCR1 affects the expression of several genes regulating cytoskeleton and cell-cell adhesion

*Tight junction* and *adherens junction*, were highlighted especially in the Iso1 cells and showed gene enrichment in the Iso3 cells as well. Interestingly we have recently observed CCHCR1 expression in the skin in the proximity of the cell membrane and desmosomes [6] that are like the tight and adherens junctions specialized for cell-cell adhesion. The related functions; *regulation of actin cytoskeleton* and *focal adhesion*, were also highlighted in the CCHCR1-HEK293 cell lines. They also showed enrichment among the mock genes. These two pathways consist of a large number of genes, hence their highlightment may result from several different gene combinations and expression changes (up versus down). *Actin cytoskeleton* and *focal adhesion* share several genes with *adherens* and *tight junctions* that were not highlighted among the mock genes. Furthermore, the functions related to the cytoskeleton are supported by our previous microscopic observation; the actin cytoskeleton of the Iso1 cells resembled that of wild type cells, but was disturbed in the Iso3 cells. Furthermore, after disruption of microtubules, the Iso3Risk cells, especially, showed aberrant actin organization [6]. Here, we showed that several relevant genes, including *TLN1* (talin 1), *FN1* (fibronectin 1), and *FLNC* (filamin C), were downregulated in the Iso3Risk cells, which may disturb cytoskeleton organization. Talin plays a role in the assembly of actin filaments and interacts indirectly with extracellular fibronectin 1 [27], and they both play a role in adhesion, spreading, and migration of cells [28, 29]. Filamin crosslinks actin filaments into networks and anchor membrane proteins to the cytoskeleton, thereby affecting cell-cell and focal adhesion. Our previous results, as well as RNAseq, also highlighted other cytoskeleton-related genes; several cytokeratin genes, e.g. KRT17 were upregulated especially in the Iso1Non-risk cells [6]. One of the most highly upregulated genes in the Iso3 cells was *SYT1*. It encodes for synaptotagmin that interacts with tubulin, and may act as a microtubule-organizing center-associated protein, thus playing a role in spindle organization [30, 31].

### CCHCR1 shows isoform-specific effects on P-body localization and P-body-related functions

We have shown that in cultured cells CCHCR1 isoforms 1 and 3 reside in the cytoplasm, as well as localize at the centrosomes [6, 8]. Interestingly, here we noticed a difference in their colocalization with the P-bodies in the HEK293 cells. The immunostaining supported colocalization with the P-bodies for isoform 3, especially at the centrosome, while isoform 1 colocalized rarely with the P-bodies. The localization of P-bodies at the centrosomes seemed to be improved by the overexpression of Iso3 CCHCR1. Recently it was shown that the N-terminus of Iso3Non-risk was necessary for the P-body localization [15], isoform 1 that is 89 amino acids longer at its N-terminus than Isoform 3 was not along in this study. The different N-terminus may explain the localization difference between isoforms 1 and 3.

Previous studies have shown that there is a strong interaction between CCHCR1 and EDC4 (enhancer of mRNA-decapping 4 protein) [15] that is a central P-body component regulating mRNA decay by decapping and 5′ → 3′ mRNA degradation. In our RNAseq, *mRNA surveillance pathway* that is detecting and degrading abnormal mRNAs [32, 33] was highlighted in the HEK293 cells expressing Iso3Non-risk, the same isoform that was used in the previous binding studies. In addition to *mRNA surveillance,* the pathway analyses of the pooled data of Non-risk and Iso3 highlighted *RNA degradation* and *RNA transport. mRNA surveillance* includes *nonsense-mediated mRNA decay* (NMD) that eliminates mRNAs containing premature translation-termination codons [33]. In the RNAseq, several *SMG* genes encoding factors of NMD surveillance complex, and *CPSF* genes encoding for cleavage and polyadenylation specific factors, were upregulated in Iso3Non-risk.

P-bodies have been associated with several autoimmune diseases, cancers, and neurological diseases [34, 35], but not with psoriasis. They are linked to viruses and innate immunity as well. The P-bodies are manipulated by the viruses for a productive infection to occur, but they also promote immune responses to the viral infection, e.g., via double-stranded RNA protein kinase (PKR alias *EIF2AK2*) [36]. Viruses, such as human papillomavirus HPV16 E6, induce in keratinocytes the localization of PKR into the P-bodies suggesting about the antiviral response. Interestingly, the interaction of CCHCR1 with HPV16 E2 induced a massive redistribution of the virus protein into cytoplasmic granules that were still co-localizing with CCHCR1 [30]. We suggest that these structures were cytoplasmic P-bodies playing an antiviral role. Interestingly, RIG-I-like receptor signaling, which was highlighted both in the CCHCR1-HEK293 cell lines and psoriatic skin, is part of the antiviral immune response. The function of RIG-I-like receptors is linked at many levels to different RNA-granules including P-bodies [37].

## Conclusions

The RNAseq highlighted pathways that support an active role for CCHCR1 in the centrosomes and P-bodies; the most significant pathways were related to the cytoskeleton and cell adhesion, and furthermore, the *mRNA surveillance* pathway was highlighted in the Iso3Non-risk cells. The centrosomes and P-bodies are physically linked to the cytoskeleton that functions as a scaffold for the P-bodies (Fig. 6) and transported mRNAs [17, 18], thus as a centrosomal P-body protein CCHCR1 might affect diverse cytoskeleton mediated processes, such as cell division, cell adhesion, and transport of mRNA and mitochondria. The RNAseq revealed clear isoform- and some haplotype-specific differences; the Iso1 and Iso3 cells showed specific clustering as two separate groups, and the Non-risk and Risk cells often exhibited opposite effects. Putative effects arising from the tumorigenic nature of HEK293 can’t be completely ruled out. In summary, the present study strengthened the previous observations about the effects and possible interaction partners of CCHCR1 in the cell, as well as provided new clues about its possible function.

## Methods

### Samples, RNA extraction, and RNAseq

Generation of stable cell lines overexpressing the different CCHCR1 isoforms was described in our previous study [6]. Briefly, CCHCR1-pDsRed-constructs (Iso1Non-risk, Iso1Risk, Iso3Non-risk, or Iso3Risk) or vector were stably transfected into HEK293 which is an easily transfectable tumorigenic cell line. Colonies resistant to G418 antibiotics were screened for the CCHCR1 expression by qPCR and fluorescence microscopy. The expression of selected clones was verified by Western blotting [6]. The RNA extraction was performed at the same time as the quantification of clones without further passaging of cell lines. For the microarray and RNAseq experiments the total RNA of the CCHCR1 cell lines and controls was extracted by a RNeasy kit (Qiagen) and RNA quality was controlled by Bioanalyzer (RIN for all samples > 8.5). Total RNA samples, three replicates for each CCHCR1-HEK293 cell line were used for the RNAseq library preparation according to the previous single-cell tagged reverse transcription (STRT) protocol [38], which was adjusted for 10 ng samples as in our previous studies [20, 24]. The libraries were sequenced using an Illumina HiSeq 2000 instrument. Pre-processing of STRT reads, alignments and per-gene quantitation were performed as previously described [38]. The data normalization and differential expression analyses were performed using SAMstrt [39] (https://github.com/shka/R-SAMstrt/wiki), which is an enhancement of SAMseq [PMID: 22127579] for the spikein-base normalization. Differentially expressed genes were extracted by two class unpaired comparison; threshold of the significantly regulated genes was FDR < 1%. We performed PCA with the scaling but non-centering pre-process steps. A higher permutation (*n* = 1000) was applied to the comparisons with pooled samples (comb_Iso1, -Iso3, −Risk, and –Non-risk). The mock analysis with permutated sample groups was performed to find possible false positive pathway results and included six manual permutations per comparison group.

The RNAseq of psoriatic skin samples was recently outlined elsewhere [20]. Briefly, all subjects involved in this study gave written informed consent and the study followed the Declaration of Helsinki Guidelines. The Institutional Review Board of the Helsinki University Central Hospital had approved the study and the collection of skin samples. The skin samples were harvested by a compressed air-driven dermatome (Zimmer®, Warsaw, IN) to obtain an epidermal sample with minimal dermis involvement. We collected lesional (PL) and non-lesional (PN) samples from six psoriatic patients and normal healthy skin from nine controls (C).

### Microarray study with isoform 1-overexpressing cells

For the microarray study we used GeneChip® Human Gene 1.0 ST Arrays. The total RNA samples of cells were hybridized according to manufacturer’s protocols (Affymetrix Inc., Santa Clara, CA, USA). The arrays were scanned with GeneChip scanner 3000 7G (Affymetrix Inc.). The analyses of the microarray data were performed using the statistical software R (http://www.R-project.org) using the Affy and Limma packages [40, 41] similar to procedure earlier described [42]. In short, we implemented the robust multiarray average method (RMA) [43] to normalize and calculate the Log2 expression values at gene level (annotated using hugene10stv1.r3cdf_2.5.0). We did pair-wise comparison contrasts between the vector control and the different CCHCR1-HEK293 cell lines followed with t-statistics and log-odds of differential expression (B-value). The original microarray data compliance with MIAME procedure has been submitted to ArrayExpress (the accession number E-MTAB-6848).

### Quantitative real-time PCR

We used qPCR to validate the expression of *AREG*, *FN1*, *TLN1*, *CXCL8,* and *SYT1* genes in the CCHCR1-HEK293 cell lines. cDNA synthesis was carried out with random primers and SuperScript III First-Strand synthesis system (Invitrogen) according to manufacturer’s instructions. qPCR was carried out using an ABI PRISM 7900HT Sequence Detection System with pre-designed TaqMan® Gene Expression Assays (Thermo Fisher Scientific) or with self-designed primers by using Fast SYBR® Green Master mix (Thermo Fisher Scientific) for detection, according to manufacturer’s instructions. Lists of pre-designed TaqMan Gene Expression Assays and nucleotide sequences of self-designed qPCR primers are shown in Additional file 8. Each sample was run in three replicates and the experiments were run twice. *HPRT1* was used as a reference gene for normalization and the samples were compared against the wild type HEK293 sample. Statistical comparisons were made with the Student’s t-test.

### SNP genotyping

We genotyped the CCHCR1 SNPs rs3130453 (CCHCR1*Iso1/3) and rs130076 (CCHCR1*WWCC/RRGS) with commercial allelic discrimination assays with pre-designed probes and primers (TaqMan) described previously [6] from DNA extracted from the skin samples [20]. Sample C.05 was not genotyped due to lack of sufficient amount of sample for DNA extraction. The HLA-Cw*06:02 genotype was also determined with commercial allelic discrimination assays (TaqMan) [44].

### Immunofluorescence

The CCHCR1-HEK293 cell lines, vector control, and wild type HEK293 cells were grown on cover slips coated with rat tail collagen I (Gibco, Invitrogen) and fixed with 4% paraformaldehyde solution. After fixation cells were permeabilized with 0.1% Triton-×100 in PBS. The samples were incubated 1 h at room temperature with the antibodies against P-body markers EDC4 (rabbit polyclonal, Cell Signaling) and DCP1A (mouse monoclonal, Abnova), and centrosome marker γ-tubulin (mouse monoclonal, Sigma). Alexa Fluor 555 and 488 conjugated IgGs (Invitrogen, Molecular Probes) were used as secondary antibodies and the nuclei stained with DAPI (4′,6-diamidino-2-phenylindole, Sigma-Aldrich). The pictures were taken with Zeiss LSM 5 Duo confocal microscope. Differences in localization of CCHCR1 isoforms with centrosomal P-bodies were determined by counting colocalized staining of CCHCR1 and EDC4 and DCP1A in each CCHCR1-overexpressing cell line blindly (Additional file 8: Supplementary Information about qPCR and co-localization of CCHCR1 with P-body markers).

## Additional files

Additional file 1: **Table S1.** Microarray data and gene enrichment analysis of the CCHCR1 Iso1 cell lines. Gene expression profiling data of microarrays from the Isoform 1 CCHCR1-HEK293 cell lines. Gene enrichment analysis of differentially expressed genes using the KEGG pathway analysis of WebGestaltR and functional cluster analysis of the DAVID. (XLSX 305 kb)

Additional file 2: **Table S2.** Differentially expressed genes of RNAseq from the comparisons between the CCHCR1-HEK293 cell lines and controls. Up- and downregulated DEGs of CCHCR1-overexpressing cell lines Iso1Non-risk (1 N), Iso1Risk (1R), Iso3Non-risk (3 N), and Iso3Risk (3R) when compared with controls (wild type and vector transfected cells). (XLSX 1215 kb)

Additional file 3: **Table S3.** Gene enrichment analysis of DEGs from the CCHCR1-HEK293 cell lines based on RNAseq. Cell line-specific gene enrichment analyses of DEGs (Additional file 2: Table S2) from the CCHCR1-overexpressing cell lines Iso1Non-risk (1 N), Iso1Risk (1R), Iso3Non-risk (3 N), and Iso3Risk (3R) when compared with wild type and vector controls. The gene enrichment analysis of DEGs that were shared by the four CCHCR1 cell lines (Intersection). Analyses were done using the KEGG pathway analysis (WebGestaltR and WebGestalt), and GO and cluster analyses from DAVID. Gene enrichment of Iso3Non-Risk DEGs in the mRNA surveillance pathway is shown (KEGG pathway figure and a list of genes). (XLSX 1183 kb)

Additional file 4: **Table S4.** Isoform- and haplotype-specific gene enrichment with the shared DEGs of the CCHCR1-HEK293 cell lines. Gene enrichment analyses of DEGs shared by only the Non-risk (Diff N), Risk (Diff R), isoform 1 (Diff iso1), or isoform 3 (Diff iso3) CCHCR1cell lines (see in detail Fig. 4 Venn diagram). The DEGs shared by all the CCHCR1 cell lines (Intersection) were analyzed as well. Analyses were done using the GO and cluster analyses from DAVID and KEGG pathway analysis from WebGestalt and WebGestaltR. (XLSX 307 kb)

Additional file 5: **Table S5.** Isoform specific gene enrichment analyses based on re-extracted DEGs of the CCHCR1-HEK293 cell lines. The DEGs were obtained from the pooled data of Iso1Non-risk and Iso1Risk, and Iso3Non-risk and Iso3Risk compared to the controls (wildtype and vector). The DEGs (comb_Iso1 and comb_Iso3) were analysed using the KEGG pathway analysis of WebGestaltR. (XLSX 3054 kb)

Additional file 6: **Table S6.** Haplotype specific gene enrichment analyses based on re-extracted DEGs of the CCHCR1-HEK293 cell lines. The DEGs were obtained from the pooled data of Iso1Non-Risk and Iso3Non-Risk, and Iso1Risk and Iso3Risk compared to the controls (wildtype and vector). The DEGs (comb_Non-Risk, comb_Risk) were analysed using the KEGG pathway analysis of WebGestaltR. Summary of the gene enrichment results among the mock DEGs lists. (XLSX 2314 kb)

Additional file 7: **Table S7** and **Figure S1.** CCHCR1 and HLA-Cw6 genotypes of the skin samples. **Figure S1.** The CCHCR1 and HLA-Cw6 genotypes illustrated in a PCA plot. (XLSX 79 kb)

Additional file 8: **Supplementary Information** and **Figure S2.** Information about qPCR and co-localization of CCHCR1 with P-body markers. Lists of pre-designed TaqMan Gene Expression Assays and nucleotide sequences of self-designed qPCR primers. Counting the colocalization of CCHCR1 with P-body markers in the CCHCR1-HEK293 cell lines and calculation of *p*-values for the comparison between cell lines. **Figure S2**. γ-tubulin staining of the CCHCR1 cells. Antibody against γ-tubulin was used as a marker for centrosomes. (PDF 1046 kb)

## Abbreviations

**Iso1*: Allele resulting in CCHCR1 isoform 1
**Iso3*: Allele resulting in CCHCR1 isoform 3
**Non-risk*: Non-risk haplotype (**RRGS*) of *CCHCR1*
**Risk*: Psoriasis-associated risk haplotype (**WWCC*) of *CCHCR1*
DEG: Differentially expressed gene
FC: Fold change
FDR: False discovery rate
Iso1 cells: CCHCR1 isoform 1 expressing cell lines
Iso3 cells: CCHCR1 isoform 3 expressing cell lines
PCA: Principal component analysis
STRT: Single-cell tagged reverse transcription
