# Supplementary material for: Intracellular signalling pathways and cytoskeletal functions converge on the psoriasis candidate gene CCHCR1 expressed at P-bodies and centrosomes
Source: BMC Genomics. 2018 Jun 4;19:432. doi: 10.1186/s12864-018-4810-y (PMC5987482; doi:10.1186/s12864-018-4810-y)
Supplement: Supplementary file 8 — Supplementary Information and Figure S2. Information about qPCR and co-localization of CCHCR1 with P-body markers. Lists of pre-designed TaqMan Gene Expression Assays and nucleotide sequences of self-designed qPCR primers. Counting the colocalization of CCHCR1 with P-body markers in the CCHCR1-HEK293 cell lines and calculation of p-values for the comparison between cell lines. Figure S2. γ-tubulin staining of the CCHCR1 cells. Antibody against γ-tubulin was used as a marker for centrosomes. (PDF 1046 kb) [file 12864_2018_4810_MOESM8_ESM.pdf]

Additional file 8: Supplementary information about qPCR and co-localization studies

qPCR studies

Pre-designed TaqMan® Gene Expression Assays:

FN1	Hs00365058_m1
TLN1	Hs00196775_m1
HPRT1	Hs01003267_m1

Sequences of self-designed qPCR primers:

SYT1	F: TGCATAAAATTCCATTGCCACCG
	R: AAAAGCAGCAGGTCAGGACT
CLCX8	F: TCTGTGTGAAGGTGCAGTTTTG
	R: TGGGGTGGAAAGGTTTGGAGT
HPRT1	F: CCCTGGCGTCGTGATTAGT
	R: CACCCTTTCCAAATCCTCAGC

Colocalization studies

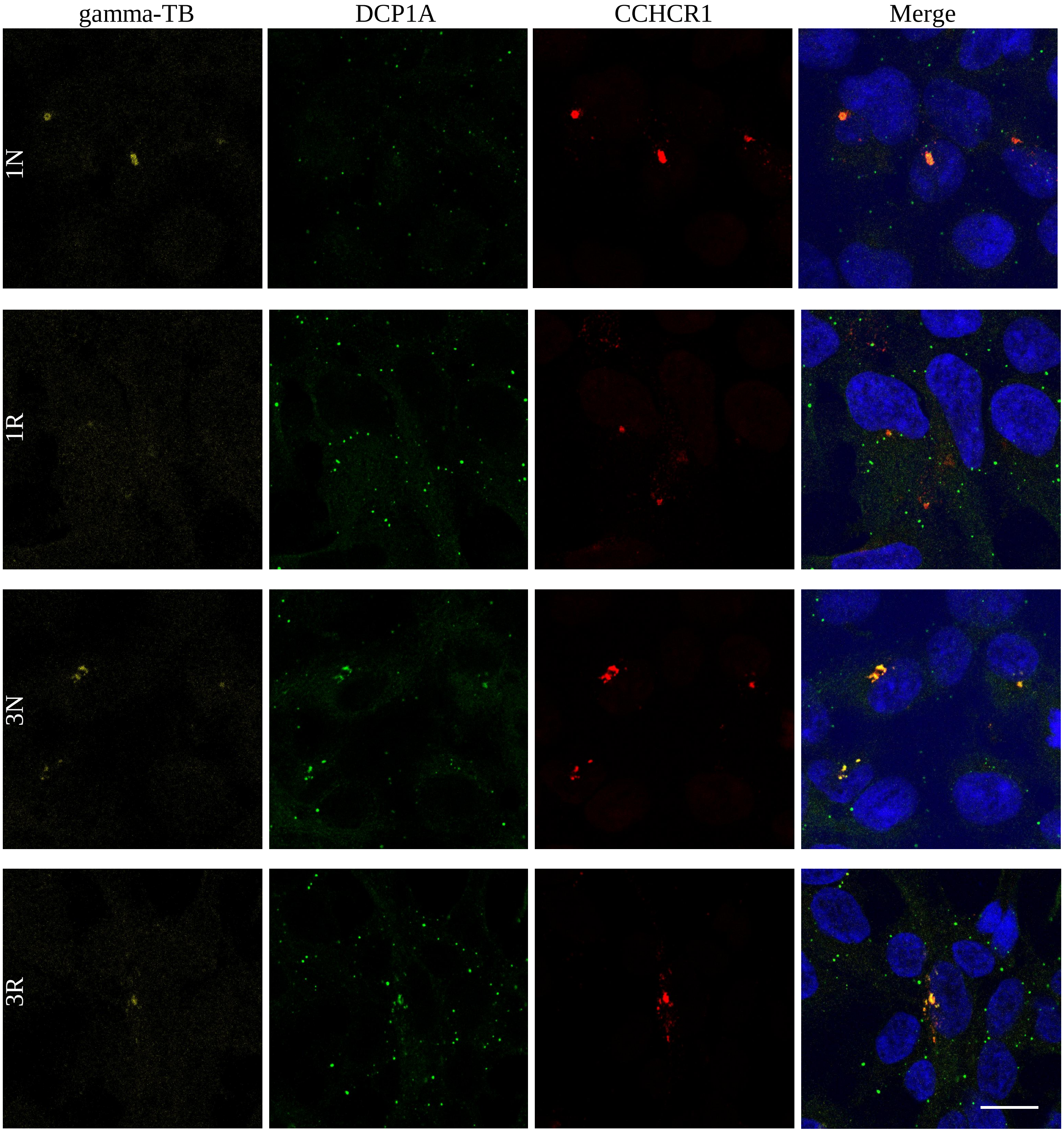

Figure S2. Colocalization of CCHCR1 with P-body and centrosome markers. Differences in localization of CCHCR1 isoforms Iso1Non-risk (1N), Iso1Risk (1R), Iso3Non-risk (3N), and Iso3Risk (3R) with centrosomal P-bodies were determined by counting colocalized (orange) staining of CCHCR1 (red) and DCP1A (green) in each CCHCR1-overexpressing cell line. Here shown the centrosome staining (gamma-tubulin, yellow). Nuclei are stained with DAPI (blue): Scale bar 10 μm.

Counting the colocalization of CCHCR1 with P-body markers in the CCHCR1 cell lines and calculation of p-values for the comparison between cell lines:

1N	1R	3N	3R		
1	0	2	2		
0	1	1	2		
0	1	2	2		
0	1	2	2		
1	0	2	2		
0	0	2	1		
0	2	2	1		
0	1	1	2		
0	0	1	1		
0	0	2	2		
0	0	2	2		
0	0	2	2		
0	1	2	2		
0	0	2	1		
0	0	2	2		
0	0	2	1		
0	0	1	2		
	0.1			1N vs 1R	p-value
		2.8E-13	3.5E-12	1N vs 3N and 3R	
		4.3E-08	1.3E-07	1R vs 3N and 3R	
		0.7		3N vs 3R	
	3.9E-19			Iso1 vs Iso3	

0 = no colocalization with CCHCR1, 1 = faint, 2 = strong. Iso1Non-risk (1N), Iso1Risk (1R), Iso3Non-risk (3N), Iso3Risk (3R).
